# Plasmacytoid Dendritic Cells Respond Directly to Apoptotic Cells by Secreting Immune Regulatory IL-10 or IFN-α

**DOI:** 10.3389/fimmu.2016.00590

**Published:** 2016-12-14

**Authors:** Joanne Simpson, Katherine Miles, Marta Trüb, Roisin MacMahon, Mohini Gray

**Affiliations:** ^1^MRC Centre for Inflammation Research, The Queen’s Medical Research Institute, University of Edinburgh, Edinburgh, UK

**Keywords:** plasmacytoid dendritic cell, apoptotic cell, apoptosis, toll-like receptor, IL-10

## Abstract

Plasmacytoid dendritic cells (pDCs) play a pivotal role in driving the autoimmune disease systemic lupus erythematosus, *via* the secretion of IFN-α in response to nuclear self-antigens complexed with autoantibodies. Apoptotic cells, generated at sites of inflammation or secondary lymphoid organs, are exposed to activated pDCs and also express the same nuclear antigens on their cell surface. Here, we show that in the absence of autoantibodies, activated pDCs directly respond to apoptotic cell-expressed chromatin complexes by secreting IL-10 and IL-6, which also induces T cells to secrete IL-10. Conversely, when activated by the viral mimetic CpG-A, apoptotic cells enhance their secretion of IFN-α. This study demonstrates that activated pDCs respond directly to apoptotic cells and may maintain tolerance *via* IL-10, or promote inflammation through secretion of IFN-α, depending on the inflammatory context.

## Introduction

During an inflammatory immune response, large numbers of tissue resident cells and leukocytes undergo programed cell death. This change from viable to apoptotic cell is potentially dangerous because neo-epitopes, including chromatin and ribonucleoprotein derived autoantigens, are expressed on the dying cell’s surface as well as on released apoptotic bodies (1–3). In addition, following infection, complexes containing viral particles and host nucleic acids are simultaneously expressed on the surface of apoptotic cells (3, 4). The consequences of losing tolerance to apoptotic cells are illustrated by the classical autoimmune diseases, systemic lupus erythematosus (SLE), and Sjogren’s syndrome that are characterized by multi-organ chronic inflammation and increased mortality (5).

However, apoptotic cells are normally highly tolerogenic, actively inducing anti-inflammatory, and immunosuppressive responses in macrophages that have phagocytosed them (efferocytosis) (6, 7). In addition, following efferocytosis, conventional dendritic cells (cDCs) present MHCII-apoptotic cell peptide complexes to cognate T cells, in the absence of costimulation, favoring T cell anergy, and the induction of immune regulation (8). Furthermore, we have previously shown that apoptotic cells interact directly with activated innate-like B cells, in a toll-like receptor (TLR) 9-dependent manner, inducing them to secrete immune regulatory IL-10 and promote self-tolerance (9, 10).

Plasmacytoid dendritic cells (pDCs) are a specialized type of dendritic cell found in steady state within the blood and secondary lymphoid organs. They also express intracellular TLR9 and TLR7 and are best known for responding to viruses by secreting copious amounts of type I IFN. To access virally infected cells, they migrate into peripheral tissues along chemokine gradients (11). Yet, pDCs have a dual role in immunity and can also promote immune tolerance (11). In particular, IFN-α secreted by pDCs induces TLR9-activated B cells to secrete IL-10 (12), which restricts autoimmune mediated inflammation (13). pDCs also promote immunological tolerance by inducing IL-10-secreting regulatory T cells (Tregs) (14–18) that may contribute to the enhanced tumor progression associated with tumor-infiltrating pDCs (19, 20). Central tolerance is also influenced by pDCs that transport peripheral antigens to the thymus and trigger thymocyte deletion (21). Additionally, immunosuppressive pDCs are found in abundance in the liver (22), where T cells undergoing activated apoptotic cell death accumulate for disposal (23).

Currently, the capacity of apoptotic cell-expressed self-antigens to directly influence the responses of pDCs is unknown. Yet, pDCs are exposed to apoptotic cells at inflamed sites, such as the cutaneous lesions associated with SLE. Here, pDCs are found to gather in inflammatory dermal regions and cluster at sites of abundant apoptotic epithelial cells (24). Aberrant stimulation of IFN-α by pDCs in response to nuclear antigens released from late apoptotic/secondary necrotic cells complexed with autoantibodies (25–27), or antimicrobial peptides (28–30) is considered to drive inflammation in SLE and Sjogren’s syndrome (11). Furthermore, pDCs accumulate in psoriatic plaques during the initiation of disease (31, 32), where they secrete IFN-α in response to self-DNA immune complexes (28). Yet, psoriasis can be successfully treated by inducing apoptosis with UVB irradiation (33), despite UVB irradiation recruiting pDCs to the skin (34).

Indeed, pDCs have been associated with the anti-inflammatory immune regulation generated during therapeutic use of apoptotic cells. Spleen-resident pDC activity is modified *in vivo* following apoptotic cell infusion, such that they acquire the ability to expand self-antigen specific Tregs (35, 36). This tolerogenic function of pDCs is required for apoptotic cell-induced facilitation of allogenic bone marrow engraftment in mice (35), and they may contribute to mediating apoptotic cell-induced protection from ongoing collagen-induced arthritis (36). However, although evidence exists that apoptotic cells can promote regulatory pDC functions *in vivo*, this was suggested to occur indirectly *via* TGF-β secretion from macrophages that had efferocytosed apoptotic cells (35).

In the clinical setting, there is potential to treat graft versus host disease (GVHD) following allogenic hematopoietic cell transplantation using extracorporeal photopheresis (ECP); a technique where peripheral blood mononuclear cells (PBMCs) are separated from whole blood, treated with 8-methoxypsoralen and exposed to UVA irradiation to induce apoptosis, then given back to the patient (37). Notably, the pDC population increased following ECP to treat patients that developed GVHD in response to stem cell transplant, indicating that pDCs may promote a favorable tolerogenic outcome (38).

Hence, activated pDCs can induce inflammation or tolerance depending on the inflammatory context (39). pDCs encounter apoptotic cells in both inflammatory and regulatory conditions, but it is not clear if apoptotic cells can directly influence their functions. pDCs endocytose antigens from infected (40) and apoptotic cells (41); again suggesting they should be able to interact with and endocytose intracellular antigens now expressed on the apoptotic cell surface. Yet, there are no studies to date that have examined if apoptotic cells can directly induce tolerogenic pDCs. In this study, we asked how pDCs might respond to apoptotic cell-expressed self-antigens, in the absence of autoantibodies or antimicrobial peptides. We find, akin to innate-like regulatory B cells, that activated pDCs do respond to apoptotic cell-expressed chromatin complexes in a TLR9-dependent manner, by secreting either IL-10 and IL-6, or IFN-α. These cytokine responses were only seen in the context of whole apoptotic cells and not debris derived from them. Activated pDCs that had been exposed to apoptotic cells also induced T cells to secrete IL-10. This indicates that activated pDCs are influenced by apoptotic cell-expressed chromatin complexes, which may contribute toward the maintenance of immune self-tolerance within an inflammatory milieu.

## Materials and Methods

### Ethical Approval

Experiments involving mice were covered by a project licence granted by the UK Home Office and approved locally by the University of Edinburgh Animal Welfare and Ethical Review Committee. Healthy donor blood was collected from the Centre for Inflammation Research blood resource approved by AMREC (Ref. 15-HV-013).

### Mice

C57BL/6 mice, C57BL/6 background TLR9^−/−^ mice, and BALB/c mice were bred and maintained in a specific pathogen-free condition in the animal facilities at University of Edinburgh in accordance to UK Home Office guidelines. TLR9^−/−^ mice were kindly provided by Prof. S. Akira (Hyogo College of Medicine, Nishinomiya, Japan). Mice were used at 6–12 weeks of age and were age- and sex-matched in experiments.

### Cell Stimulation and Treatments

Cells were treated with the following: TLR7 ligand R848, 1 µg/ml (InvivoGen); mouse TLR9 ligands, CpG-A, 20 µg/ml (ODN 1585, InvivoGen) and CpG-B, 10 µg/ml (ODN 1826, Eurofins MWG Operon); human TLR9 ligands CpG-A, 3 µg/ml (ODN 2216, InvivoGen) and CpG-B, 2 µM (ODN 2006, Eurofins MWG Operon); and DNase, 50 µg/ml (Roche, UK).

### pDC Isolation and Culture

Mouse pDCs were enriched from single-cell splenic suspensions following initial depletion of B cells using CD19 microbeads (Miltenyi Biotec). pDCs (PDCA^+^ B220^+^ Ly6C^+^ CD3^−^ CD11b^−^ CD19^−^) were further sorted using a FACSAria cell sorter (BD Biosciences) to generate >99% pure (PDCA1^+^ Ly6C^+^) pDC population (Figure S1A in Supplementary Material). pDCs (1 × 10^4^) were cocultured with apoptotic thymocytes (1 × 10^6^), or apoptotic splenic B cells (2 × 10^5^) and, where stated, splenic T cells (1 × 10^5^) isolated using CD4 microbeads (Miltenyi Biotec), in 96-well round bottom plates (Corning). Cultures were maintained in IMDM supplemented with 10% FCS, 2mM l-glutamine, 100 U/ml penicillin, 100 µg/ml streptomycin, and 2 µM 2-mercaptoethanol (complete IMDM) at 37°C 5% CO_2_ for the duration of the assay. In transwell experiments, pDCs (4 × 10^4^) were cultured in the well and apoptotic cells (4 × 10^6^) located in the upper transwell insert (permeable membrane 0.4 µM pore size) in 24-well plates (Corning).

Peripheral blood mononuclear cell (PBMC) were separated from the blood of healthy donors using dextran sedimentation (Pharmacosmos) followed by a percoll gradient (GE Healthcare) as described previously (42), with modifications. PBMC was depleted of B cells using CD19 microbeads (Miltenyi Biotec). CD19^−^ PBMC (1 × 10^6^) was cocultured with CD4^+^ apoptotic cells (1 × 10^6^) in 96-well round bottom plates (Corning) in RPMI supplemented with 10% FCS, 2mM l-glutamine, 100 U/ml penicillin, and 100 µg/ml streptomycin.

### Generation of Apoptotic Cells

Single-cell suspensions of murine thymocytes, splenic B cells purified using CD19 microbeads (Miltenyi Biotec), and human CD4^+^ cells purified from PBMCs [using CD4 microbeads (Miltenyi Biotec)] were exposed to 100 mJ/cm^2^ UV irradiation then incubated for 4 h in complete medium at 37°C 5% CO_2_. In certain experiments, DNase was added to complete medium. Apoptotic cell membranes were disrupted by performing five cycles of freezing on dry ice and thawing in 37°C water bath. The proportion of apoptotic thymocytes was measured by flow cytometry by staining cells for DNA using propidium iodide (PI) and surface expression of phosphatidylserine using Annexin V-FITC (Invitrogen). The minimum degree of apoptosis for cells used in coculture experiments was 20% early apoptotic and 15% late apoptotic. The proportion of apoptotic B cells was measured by flow cytometry using PI and 100 nM 10-*N*-nonyl acridine orange as a marker of mitochondrial membrane integrity.

### Cytokine Quantification

The concentration of mouse IL-10, IL-6, IL-12, and TNF-α was measured using standard ELISA (R&D Systems). VeriKine mouse IFN-α ELISA kit was used to quantify the concentration of IFN-α in accordance with the manufacturer’s protocol (PBL Interferon Source). Human IFN-α was quantified by matched antibody pairs in accordance with the manufacturer’s instructions (eBioscience).

### Intracellular IL-10 Cytokine Staining

Cells were taken on day 3 of culture and resuspended at 1 × 10^6^ cells/ml in fresh medium with added PMA, 20 ng/ml (Sigma-Aldrich) and Ionomycin, 1 µg/ml (Sigma-Aldrich). After 1 h, brefeldin A, 1 µg/ml (Sigma-Aldrich) was added to block secretion of cytokines and the cells were incubated for an additional 3 h. The cells were then surface-stained (PDCA1-APC and B220-PerCP), followed by fixation and permeabilization (BD Biosciences) before intracellular cytokine staining with PE rat anti-mouse IL-10 (1:100; BD Pharmingen) or PE rat IgG2b isotype control (1:100; BD Pharmingen).

### IL-10 Secretion Assay

The mouse IL-10 secretion assay was performed in accordance to the manufacturer’s instructions (Miltenyi Biotec) with a few minor changes. Briefly, on day 7 of culture, cells were washed and resuspended in complete mouse medium containing IL-10 catch reagent and incubated on ice for 5 min. The cells were then continuously rotated for 4 h at 37°C prior to washing and staining with PE-conjugated mouse IL-10 detection antibody (Miltenyi Biotec) and anti-mouse CD4-FITC (1:100; BioLegend).

### Flow Cytometry

Single stain controls of each fluorochrome were used to determine appropriate compensation. Samples were selected using singlet gating and were obtained by FACS Calibur or LSR Fortessa (BD Biosciences) and analyzed using FlowJo Software (version 10.0).

### Immunizations

The 5 × 10^6^ DO11.10 lymph node cells were injected i.v. into BALB/c mice followed by immunization with OVA_323–339_ peptide (2 µg/ml) emulsified in CFA s.c. into each hind leg. Then 20 × 10^6^ apoptotic thymocytes or vehicle alone (PBS) were injected i.v. on days 0, 1, and 2. On day 7, single-cell suspensions of spleen and draining (inguinal) lymph nodes were flow sorted to obtain purified pDCs and CD11b^+^ CD11c^+^ cDCs. pDCs and cDCs (10^4^) were cultured for 72 h with CD4^+^ DO11.10 T cells (10^5^) and OVA_323–339_ peptide (2 µg/ml).

### Statistical Analysis

Data are expressed as mean and SEM. Statistical significance between the groups was assessed by GraphPad Prism Version 7.0 using the appropriate analysis as stated in the figure legends. *p* Values: *0.01–0.05, **0.001–0.01, and ***<0.001.

## Results

### pDCs Exposed to Apoptotic Cells *In Vivo* Augment IL-10-Secreting Ovalbumin (OVA)-Specific CD4^+^ T Cells

**Figure 1:**
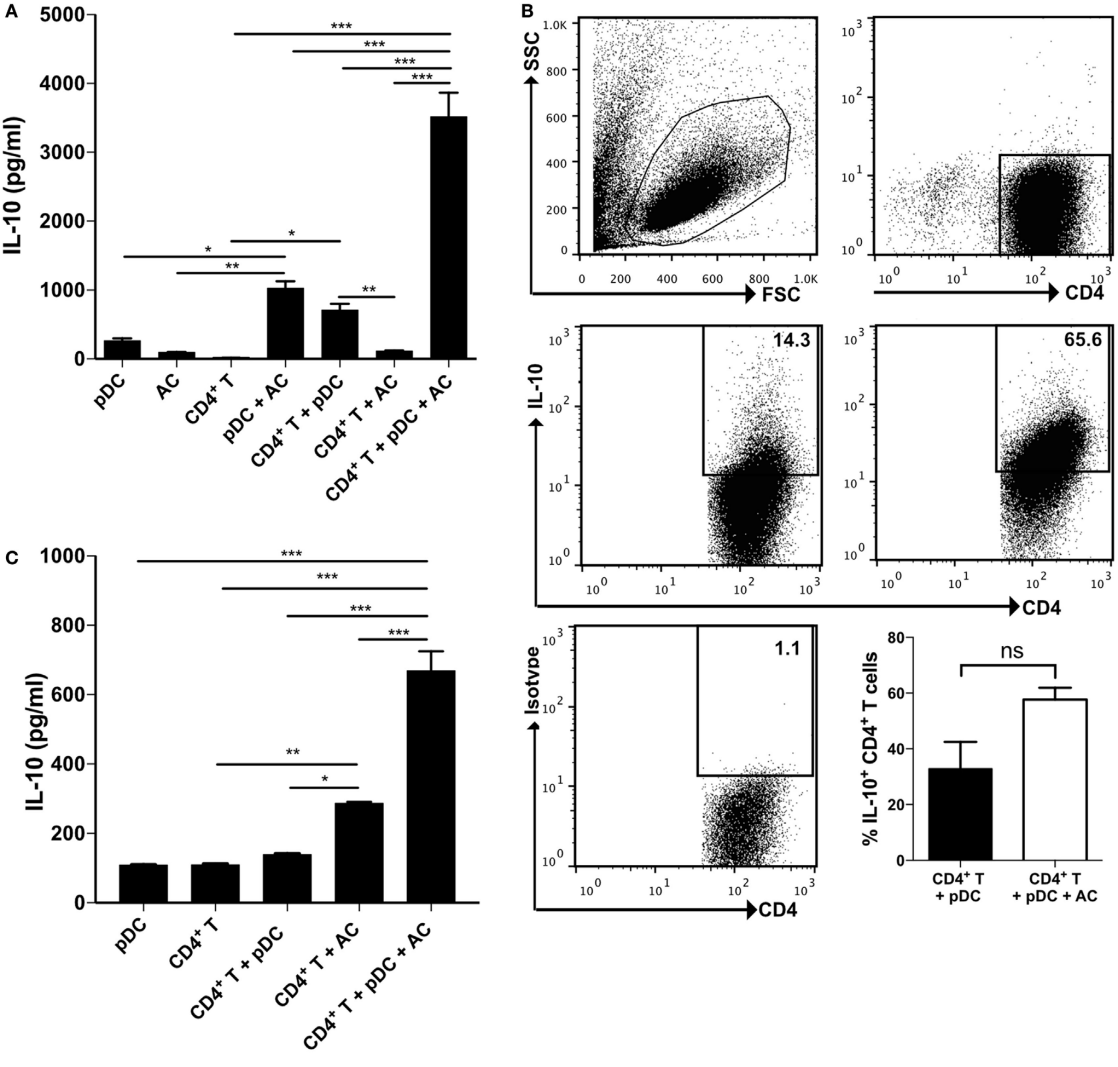

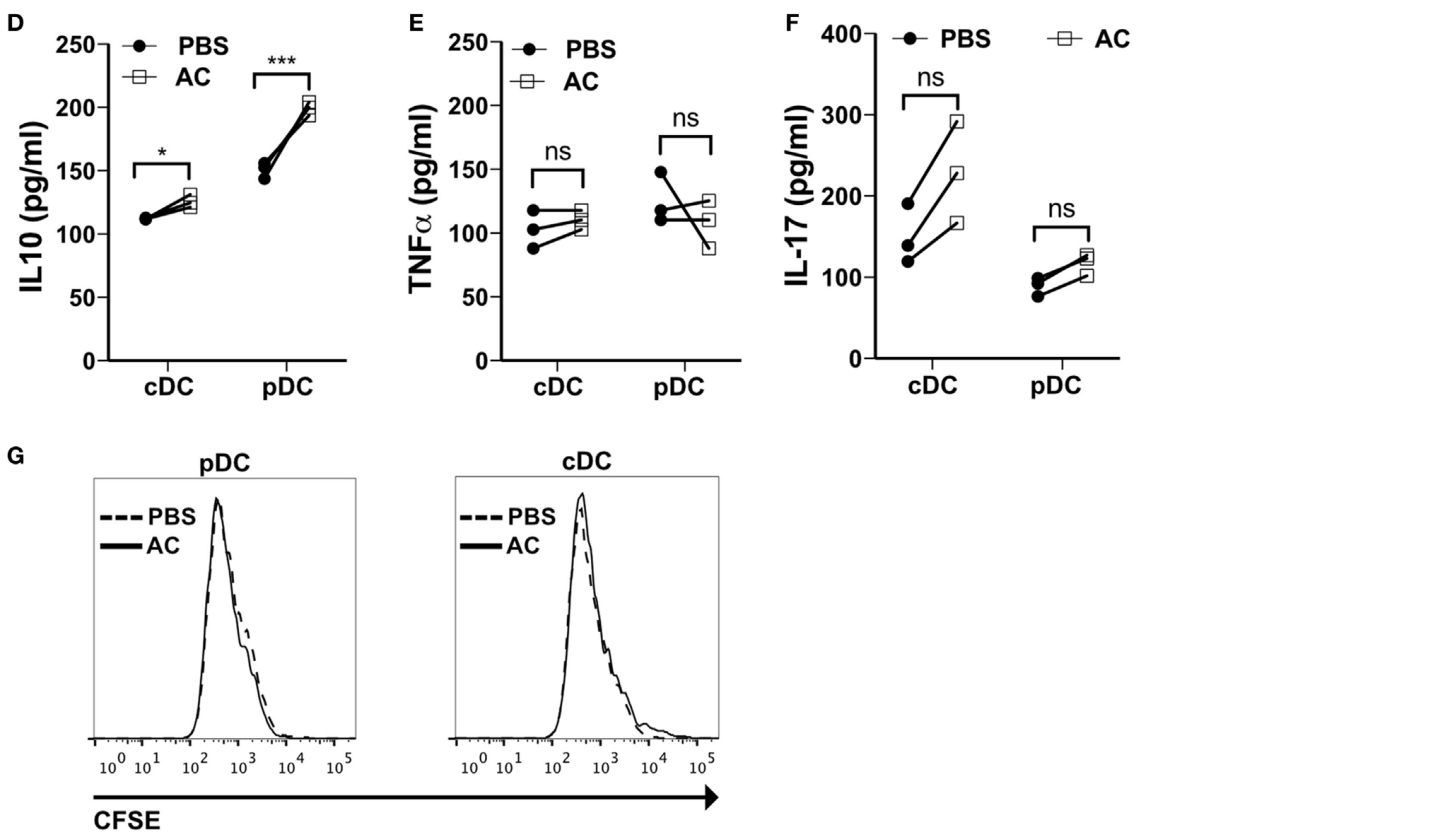
**Plasmacytoid dendritic cells (pDCs) that have encountered apoptotic cells *in vivo* induce a population of IL-10 secreting CD4^+^ T cells**. **(A)** CD4^+^ T cells were cultured with R848, pDCs, and apoptotic B cells in various combinations. After 7 days, IL-10 production was measured by ELISA. Data are shown as the mean + SEM of three independent experiments. *p < 0.05; **p < 0.01; and ***p < 0.001, as determined by one-way ANOVA followed by Tukey’s multiple comparison test. **(B)** The proportion of CD4^+^ T cells producing IL-10 in response to R848 and pDCs with and without apoptotic B cells was quantified by IL-10 secretion assay. Data are shown as the mean + SEM of three independent experiments, with representative FACS plots from one of three experiments. **p < 0.01, as determined by Student’s *t*-test. **(C)** BALB/C CD4^+^ T cells were cultured with R848, C57BL/6 pDCs, and C57BL/6 apoptotic B cells in various combinations. IL-10 production was measured after 3 days. *p < 0.05; **p < 0.01; and ***p < 0.001, as determined by one-way ANOVA followed by Tukey’s multiple comparison test. **(D)** IL-10, **(E)** TNF-α, and **(F)** IL-17 production by naïve DO11.10 CD4^+^ T cells was measured following 72 h in culture with OVA_323–339_ peptide and pDCs, or conventional dendritic cells (cDCs) that were isolated from the spleen and inguinal lymph nodes of mice that were immunized for 7 days with OVA/CFA and vehicle control (PBS), or apoptotic thymocytes (AC). Each symbol represents pooled data from three independent experiments consisting of four mice per group. *p < 0.05; ***p < 0.001; and ns not significant, as determined by paired Student’s *t*-test. **(G)** DO11.10 CD4^+^ T cell proliferation was measured following 72 h in culture with OVA_323–339_ peptide and pDCs, or cDCs. FACS plots represent data pooled from four mice. See also Figure S1C in Supplementary Material.

We have previously shown that apoptotic cells, administered at the time of inducing an antigen specific immune response with OVA peptide specific T cells, were able to induce the secretion of IL-10 by innate-like B cells, present in the spleen and peritoneal cavity (9, 10). We hypothesized that apoptotic cells could equally drive activated pDCs to adopt a regulatory phenotype, capable of inducing IL-10-secreting T cells. To test this, we cocultured pDCs that had been activated through TLR7 (with R848) along with apoptotic B cells and CD4^+^ T cells for 7 days. This resulted in a significant increase in IL-10 in the culture medium (Figure 1A). Cytokine secretion staining confirmed that, while TLR7-activated pDCs induced 14% of T cells to secrete IL-10, this was augmented fourfold in the presence of apoptotic cells (Figure 1B; Figure S1B in Supplementary Material). Similar results were seen when TLR7-activated pDCs, cultured with apoptotic B cells were used as APCs in a mixed lymphocyte reaction along with allogeneic T cells (Figure 1C).

The location of pDCs within the spleen and lymph nodes increases the likelihood that they will interact with apoptotic cells, filtered from the circulation or dying *in situ*. We next compared the responses of pDCs and cDCs (CD11b^+^ CD11c^+^) purified from the spleen and draining lymph nodes of mice that had been immunized with OVA/CFA and injected intravenously with apoptotic thymocytes or with vehicle control (PBS). pDCs and cDCs, pulsed with OVA peptide, were then used as APCs to stimulate naïve OVA-specific T cells with OVA peptide *in vitro*. pDCs isolated from the same apoptotic cell-treated mice, induced a higher concentration of IL-10 than cDCs (Figure 1D). In addition, those cultures containing pDCs isolated from apoptotic cell-treated mice generated significantly more IL-10, than pDCs isolated from PBS-treated mice (Figure 1D). In contrast, the production of TNF-α and IL-17 was not significantly different (Figures 1E,F). The proliferation of OVA-specific T cells was equivalent, when both pDCs and conventional cDCs (Figure 1G) served as the APC and T cells did not express more FoxP3 (Figure S1C in Supplementary Material). These data suggest that in a similar way to regulatory B cells (9), apoptotic cells influence the ability of activated pDCs to induce the generation of IL-10 in cultures with naïve T cells.

### The pDC Response to Apoptotic Cells Depends on the Coactivating Stimulus

**Figure 2:**
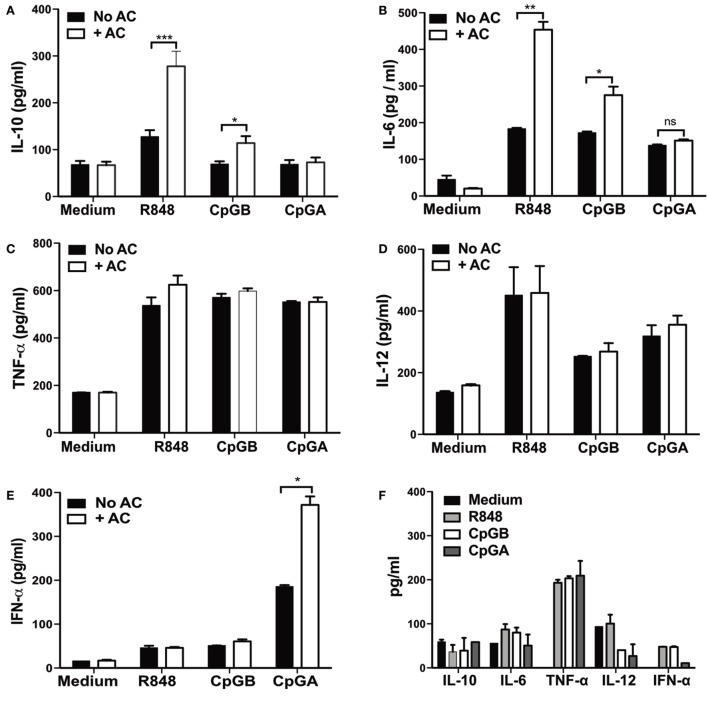

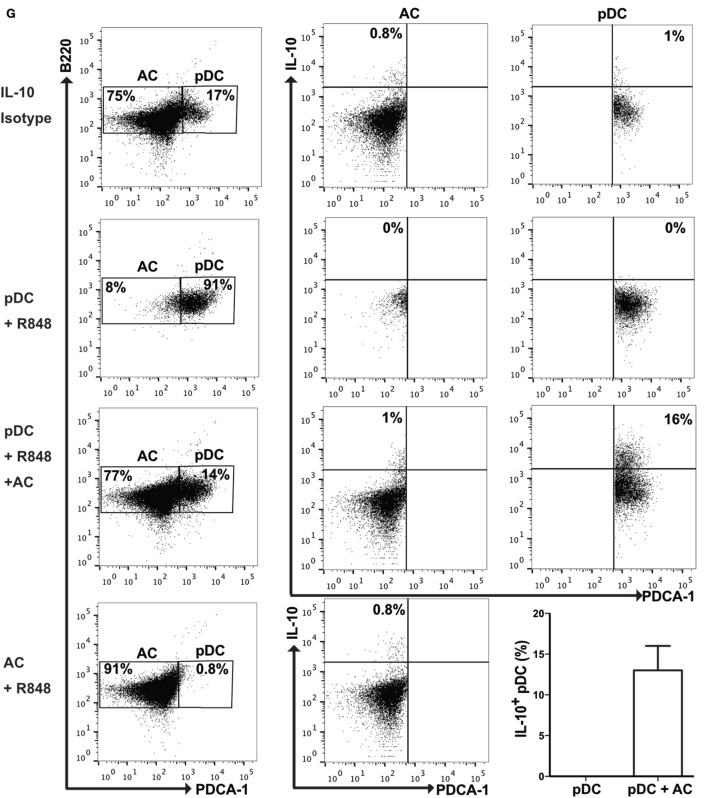
**Apoptotic cells augment differential cytokine production by plasmacytoid dendritic cells (pDCs) depending on the toll-like receptor stimulus**. pDCs were unstimulated, or stimulated with R848, CpG-B, or CpG-A, and cultured alone (No AC), or with apoptotic thymocytes (+ AC). **(A)** IL-10, **(B)** IL-6, **(C)** TNF-α, **(D)** IL-12, and **(E)** IFN-α protein were measured in cell supernatants after 72 h. **(F)** Cytokine production by apoptotic thymocytes cultured alone with and without R848, CpG-B, or CpG-A was measured after 72 h. Data are presented as the mean of three to eight independent experiments. Error bars represent SEM. *p < 0.05; **p < 0.01; ***p < 0.001; and ns not significant, as determined by paired Student’s *t*-test. **(G)** Expression of intracellular IL-10 was detected in pDCs (PDCA^+^ B220^+^) that were cultured for 72 h with R848 alone and with apoptotic B cells (+ AC), and apoptotic B cells with R848 alone. FACS plots represent one of three independent experiments. Bars are the mean + SEM of three independent experiments. See also Figures S1A,D–H and S2 in Supplementary Material.

To begin to dissect the requirements for apoptotic cell-mediated augmentation of cytokine secretion by pDCs, we first asked if resting pDCs could respond directly to apoptotic cells. In the absence of other stimuli, 10^4^ highly purified pDCs (Figure S1A in Supplementary Material) were cocultured for 72 h with 10^6^ apoptotic thymocytes. Despite one-third of the apoptotic cells becoming late apoptotic within 24 h (Figure S1D in Supplementary Material), they failed to secrete any of the cytokines tested, including IL-10, IL-6, TNF-α, IL-12, or IFN-α (Figures 2A–E). This is likely due to the failure of apoptotic cells alone to activate pDCs, which is supported by the lack of CD86, CD40, or MHC class II upregulation (Figure S1D in Supplementary Material) and also by their reduced viability (Figure S1G in Supplementary Material). Hence in the absence of autoantibodies, resting pDCs do not respond to apoptotic primary cells, even when the apoptotic cells have become late apoptotic.

To mimic the presence of single-stranded RNA viruses and to provide an activating stimulus to the pDCs, the TLR7 ligand R848 was included in the coculture with apoptotic thymocytes. pDCs were activated, as indicated by the increased expression of CD86 and MHC Class II (Figure S1F in Supplementary Material) and also showed enhanced survival over 72 h (Figure S1F in Supplementary Material). Within 48 h, in the presence of apoptotic cells, TLR7-activated pDCs were seen to secrete significantly more IL-10 and IL-6 (Figures 2A,B), which was maximal after 72 h (Figure S1H in Supplementary Material); but not TNF-α, IL-12, or IFN-α (Figures 2C–E). A similar pattern of cytokine secretion was seen when pDCs were activated through the TLR9 receptor with CpG-B (Figures 2A–E), which was also able to bind to the surface of apoptotic cells (Figure S2A in Supplementary Material). In contrast, when pDCs were activated with the TLR9 ligand CpG-A, and cultured with apoptotic cells [which also bound to apoptotic cells (Figure S2B in Supplementary Material)], the release of IFN-α (Figure 2E) [but not other measured cytokines (Figures 2A–D)] was significantly enhanced. The secretion of cytokines by apoptotic thymocytes cultured with the TLR agonists themselves (in the absence of pDCs) was minimal (Figure 2F). This indicates that in contrast to resting pDCs, TLR-activated pDCs respond to apoptotic cells. The coactivating TLR stimulus (which may mimic viral particles bound to the surface of apoptotic cells), determined the subsequent cytokine profile of cytokines secreted by pDCs in response to apoptotic cells. As previously reported (43), we did not detect IL-10 (by intracellular staining) in pDCs activated *via* TLRs alone (Figure 2G). However, IL-10 was clearly seen in 16% of TLR-activated pDCs following coculture with apoptotic B cells (Figure 2G; Figure S2C in Supplementary Material). Intracellular IL-10 was not detected in the apoptotic B cells, confirming that the pDC and not the apoptotic cell was the source of IL-10 (Figure 2G). Along with the *in vivo* observations, these data indicate that apoptotic cells modulate highly purified pDC function such that they secrete significantly more cytokine, which is influenced by the simultaneous activating signal; provided either by T cells or TLR ligands.

### Murine and Human pDCs Respond to Whole Apoptotic, but Not Secondary Necrotic Cells

**Figure 3:**
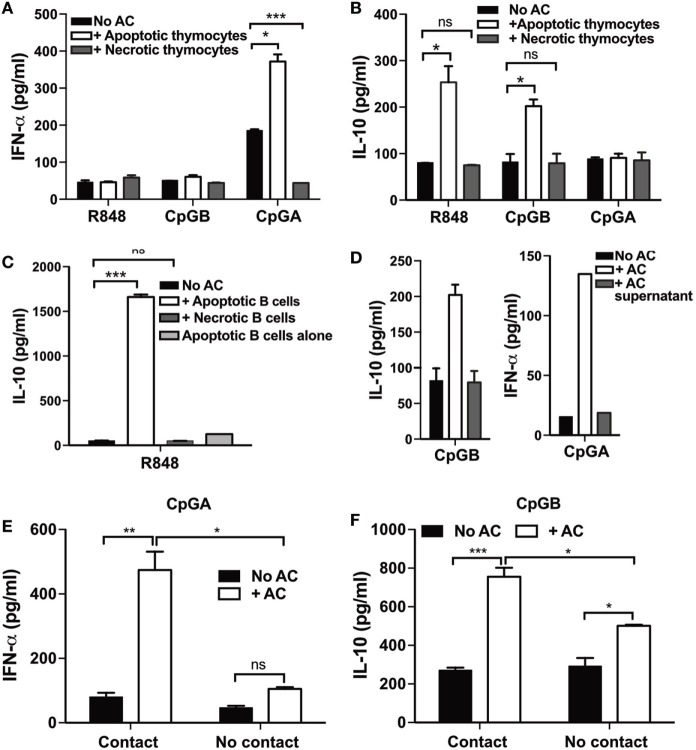
**Direct contact with whole apoptotic cells, but not necrotic cells, induces cytokine production by plasmacytoid dendritic cells (pDCs)**. **(A)** IFN-α and **(B)** IL-10 production was measured 72 h after R848-, CpG-B-, or CpG-A-stimulated pDCs were cocultured with apoptotic, or secondarily necrotic thymocytes. **(C)** IL-10 production by R848-stimulated pDCs was measured 3 days after culture alone and with apoptotic, or secondarily necrotic B cells. IL-10 secretion by R848-stimulated apoptotic B cells alone was also measured. **(D)** pDCs activated by CpG-B and CpG-A were cultured for 72 h with the cell-free supernatant from apoptotic thymocytes and then IL-10 and IFN-α production was measured, respectively. **(E)** IFN-α and **(F)** IL-10 secretion was quantified 72 h following the coculture of CpG-A and CpG-B, respectively, activated pDCs with apoptotic cells together in the well (contact) or separated using a transwell insert (No contact). Data are shown as the mean of three independent experiments with error bars representing SEM. *p < 0.05; **p < 0.01; ***p < 0.001; and ns not significant, as determined by one-way ANOVA **(A–D)** and two-way ANOVA **(E–F)**.

Plasmacytoid dendritic cells are known to secrete IFN-α in response to immune complexes formed between SLE Ig and nuclear material released from necrotic cell lines, but not apoptotic primary cells (25). Yet, SLE is caused by defective efferocytosis resulting in apoptotic cells becoming secondary necrotic (44). We therefore wished to understand how apoptotic cells were able to augment cytokine secretion by activated pDCs in the absence of autoantibodies. We asked if debris derived from primary apoptotic cells could be responsible for augmenting IFN-α secretion by CpG-A-activated pDCs. In contrast to whole apoptotic cells (thymocytes), debris released from secondarily necrotic cells did not enhance IFN-α production by CpG-A-stimulated pDCs (Figure 3A). IL-10 production by R848- or CpG-B-activated pDCs was similarly abolished if the apoptotic cells were first broken apart by repeat freeze-thawing (Figure 3B). To determine, if this effect was specific to apoptotic thymocytes, R848-activated pDCs were also cocultured with apoptotic and secondary necrotic splenic B cells, which yielded similar results (Figure 3C). This shows that, in the absence of autoantibodies, TLR-activated pDCs are responding to ligands expressed on the surface of the intact apoptotic cell membrane and not simply soluble nuclear antigens released from secondary necrotic cells.

Apoptotic cell-derived membrane microparticles have been reported to stimulate the production of IFN-α secretion by human pDCs (45). We went on to assess if TLR-activated mouse pDCs were responding to soluble factors released by apoptotic cells. Following an overnight culture of 10^6^ apoptotic thymocytes, supernatant was transferred to TLR-activated pDCs, which were cocultured for an additional 72 h. Again, apoptotic cell supernatants did not augment IL-10, or IFN-α production by CpG-B- and CpG-A-activated pDCs, respectively (Figure 3D). Furthermore, inhibiting cell-to-cell contact by separating pDCs and apoptotic cells in culture using a transwell insert significantly reduced both apoptotic cell-induced IFN-α (Figure 3E) and IL-10 (Figure 3F) production by pDCs stimulated with CpG-A and CpG-B, respectively. This indicates that activated mouse pDCs do not secrete cytokines in response to components, such as microvesicles or endosomes, released into the culture medium by the apoptotic cells. In addition, cytokine release by pDCs requires contact with the whole apoptotic cells.

### IL-10 Secretion by pDCs Is Dependent on Apoptotic Cell-Expressed Chromatin Complexes

**Figure 4:**
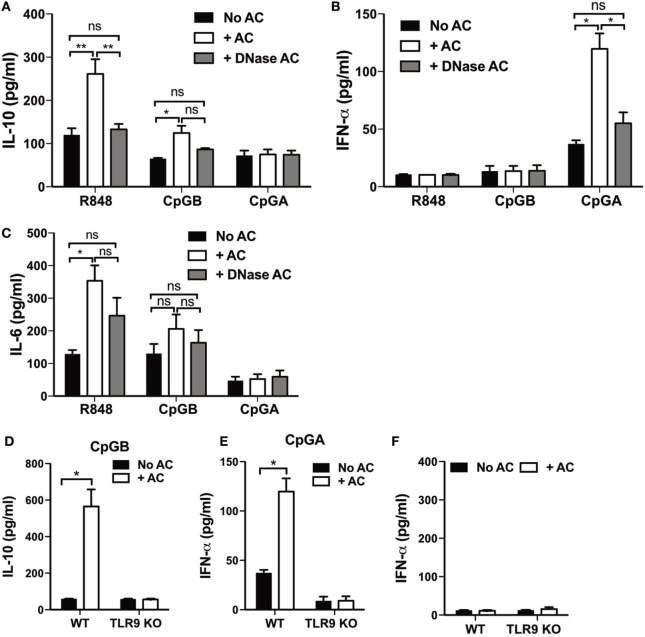
**IL-10 production by plasmacytoid dendritic cells (pDCs) is dependent on apoptotic cell-derived DNA complexes**. **(A)** IL-10, **(B)** IFN-α, and **(C)** IL-6 production was measured 72 h after pDCs were cultured with R848, CpG-B, or CpG-A, in the presence and absence of apoptotic thymocytes (+ AC), or DNase-treated apoptotic thymocytes (+ DNase AC). Data are shown as the mean + SEM of three to eight independent experiments. *p < 0.05; **p < 0.01; and ns not significant, as determined by one-way ANOVA followed by Tukey’s multiple comparison test. **(D)** IL-10 and **(E)** IFN-α secretion was quantified following culture of pDCs isolated from wild type C57BL/6 and TLR9-deficient (TLR9 KO) mice with CpG-B and CpG-A, respectively, with and without apoptotic thymocytes. Data are the mean + SEM of **(D,E)** eight and **(F)** three independent experiments. *p < 0.05, as determined by paired Student’s *t*-test.

Apoptotic cells express nuclear DNA-containing chromatin complexes on the cell surface and released apoptotic bodies (1). We have previously shown that apoptotic cell-derived DNA complexes are essential for apoptotic cell-mediated induction of IL-10-secreting regulatory B cells (10). To establish if chromatin containing complexes, expressed on apoptotic cells, were also important for inducing cytokine secretion from TLR7/9-stimulated pDCs, DNA was enzymatically removed from the apoptotic thymocyte surface using DNase (10). While this did not affect the ability of CpG-B and CpG-A to bind to the surface of the apoptotic cells (Figures S2A,B in Supplementary Material), the production of IL-10 and IFN-α (but not IL-6) from the combined apoptotic cell/TLR-stimulated pDCs was now markedly reduced (Figures 4A–C). This indicates that in order for activated pDCs to respond to apoptotic cells by upregulating the secretion of IL-10 or IFN-α, they must first be exposed to endogenous nucleic acid containing complexes, presented to the pDCs on an intact membrane. TLR9 expressed by innate-like B cells plays an essential tolerogenic role, inducing regulatory B cells in response to apoptotic cell-derived DNA (10), as well as influencing pDC-dependent T cell regulation (46). Therefore, we were interested to determine if IL-10 production by pDCs required TLR9. As expected, IL-10 and IFN-α secretion were not detected in TLR9-deficient pDCs cocultured with apoptotic thymocytes and TLR9 ligands CpG-B and CpG-A, respectively (Figures 4D–F).

**Figure 5:**
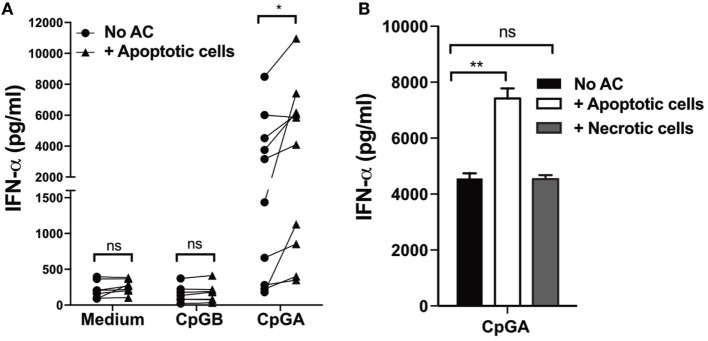
**IFN-α production by human plasmacytoid dendritic cells (pDCs) stimulated with CpG-A is enhanced by apoptotic cells, but not secondary necrotic cells**. **(A)** Peripheral blood mononuclear cells (PBMCs) isolated from healthy volunteers were cultured for 72 h with and without apoptotic cells in culture medium alone, or in the presence of CpG-B, or CpG-A, after which IFN-α in the supernatants was measured. Each symbol represents an individual healthy blood donor and data were collected from at least 10 healthy donors. *p < 0.05 and ns not significant, as determined by paired Student’s *t*-test. **(B)** IFN-α in supernatants was measured 72 h after PBMCs were stimulated with CpG-A and cultured alone (No AC) and with apoptotic, or necrotic CD4^+^ T cells. Data are representative of three independent experiments, with error bars indicating SEM. **p < 0.01 and ns not significant, as determined by one-way ANOVA followed by Tukey’s multiple comparison test.

Finally, we assessed if human pDCs would also respond to apoptotic cells in a similar way to mouse pDCs. Human pDCs and B cells are the only peripheral blood cells known to constitutively express both TLR7 and TLR9. Within the mixed populations of (neutrophil depleted) leukocytes present in PBMCs, CpG-A stimulates IFN-α production exclusively by human pDCs (47). To remove the only other source of TLR9, we depleted B cells from the PBMC population and stimulated the remaining leukocytes with CpG-A in the presence or absence of apoptotic CD4^+^ T cells. As previously noted, in the absence of additional TLR ligands, apoptotic cells cocultured with pDCs did not induce IFN-α secretion (Figure 5A). However, they significantly enhanced pDC secretion of IFN-α protein in the presence of CpG-A (Figure 5A). As before with mouse pDCs, the increase in IFN-α secretion did not occur in the presence of necrotic debris (Figure 5B), suggesting that both human and mouse pDCs are dependent on whole apoptotic cells to enhance the secretion of IFN-α.

## Discussion

Apoptotic cells are likely to make contact with pDCs within inflamed tissues following anti-viral type I IFN induced cell death, lymphoid organs (including the marginal zone of the spleen) and normal tissues such as the thymus. Yet, the ability of resting and activated pDCs from healthy subjects to directly respond to apoptotic cells has not been clearly defined. We found that an intravenous infusion of apoptotic cells prompted splenic pDCs to adopt a regulatory phenotype. When isolated from the spleen, these pDCs induced naïve T cells to secrete IL-10 *in vitro*. This study confirms that resting pDCs do not regard apoptotic primary cells or necrotic debris derived from them as a danger signal, even following prolonged coculture. However, depending on the coactivation stimulus, intracellular self-antigens expressed on the surface of whole apoptotic cells, augmented either IL-10/IL-6 or IFN-α secretion by pDCs. pDC activation following T cell stimulation or *via* TLR7 and TLR9 (by R848 and CpG-B, respectively), induced pDCs to secrete significantly more IL-10 in response to coculture with apoptotic cells. To our knowledge, this is the first time that activated pDCs have been reported to secrete IL-10 in response to apoptotic cells; though these stimuli have been shown to induce IL-10 by regulatory B cells in a TLR9-dependent manner (10). It also indicates that pDCs can respond to apoptotic cell-expressed nuclear complexes when they receive a second activating signal; provided either by a TLR ligand or activated T cells.

Plasmacytoid dendritic cells also play an important role in influencing adaptive immune responses and are ideally placed to interact with T cells in secondary lymphoid organs. They have been shown to lessen the severity of autoimmune mediated arthritis and asthma in mice (48, 49). Human pDCs can also ingest microvesicles from apoptotic cells and present the antigen to T cells (50). In the presence of apoptotic cells, TLR7 activated pDCs greatly augmented T cell expression of IL-10, without affecting T cell proliferation. Thus, the ability of activated pDCs to secrete IL-10 in the presence of apoptotic cells may help to preserve self-tolerance to dying cells within an inflammatory milieu.

Activated pDCs required direct contact with whole apoptotic cells to elicit optimal cytokine responses, but failed to respond to debris derived from necrotic cells. DNase treatment of the apoptotic cells, which stripped mammalian DNA from complexes expressed on the apoptotic cell membrane (10), also abolished the pDC cytokine responses, without affecting the binding of CpG-A or CpG-B to the apoptotic cell itself. Enzymatic digestion by DNase may break apart vital interactions between DNA and other nuclear components and prevent pDC induced cytokine responses. For example, the DNA-binding protein HMGB1, which becomes oxidized during apoptosis, has been shown to be important for inducing tolerance to apoptotic cells (50), and it is known to be expressed in membrane vesicles released by apoptotic cells (51).

Toll-like receptor 9 expression by pDCs was necessary for the augmented cytokine responses to dying cells, likely responding to the cell-expressed nuclear complexes. In humans, TLR9 activation of pDCs by CpG-B induces Tregs (46) and they also promote tolerance in a model of cardiac allograft transplantation, where they present donor derived antigens to induce Tregs (52). A regulatory role for TLR signaling has also been reported in TLR9-deficient lupus-prone mice. Here, despite the reduction in anti-DNA antibody titers, mice suffer from more severe organ damage, akin to human SLE (53–57). Hence, TLR9 expressed by activated pDCs may help to prevent autoimmune responses to apoptotic cells, through the secretion of IL-10.

We also noted that, in the absence of autoantibodies, CpG-A-activated pDCs also secreted significantly more IFN-α when cultured with whole apoptotic cells. Previous reports indicate that high affinity IgG in complex with self-antigens (released from necrotic cell lines), induce pDCs to secrete IFN-α (25, 58), so driving chronic inflammation in patients with SLE; through the activation of multiple innate and adaptive immune cells (59). However, IFN-α is also reported to have immunomodulatory effects. For example, one of the benefits of IFN treatment for patients with multiple sclerosis is believed to include the induction of IL-10 secretion (60), with IFN additionally increasing the sensitivity of monocytes to IL-10 (61). IFN-α also enhances the TLR7/8 induced production of IL-10 by human B cells (62), which dampens both innate and adaptive immune responses. It is established that viral antigens and mammalian nucleic acid complexes co-cluster on the cell surface (4); making it possible that the stimuli provided by the synthetic TLR ligands mimic the effect of viral antigens coexpressed on dying cells with self-antigens. If so, we would hypothesize that pDCs would respond optimally when these two key elements are present on the same intact membrane.

We were unable to visualize whole apoptotic cells within pDCs, and currently it is not clear how chromatin complexes expressed on apoptotic cells gain access to endosomes. However, as immature pDCs can endocytose antigens (40), they should be able to interact with and endocytose chromatin complexes expressed by apoptotic cells (41). It also calls into question the need for TLR7, 8, and 9 to exist in intracellular compartments, to prevent pDCs from being exposed to apoptotic cell-expressed nucleic acid complexes or free endogenous nucleic acids (63). It is just as likely that TLRs traffic to endosomes as a means to function optimally, rather than as a mechanism to avoid interacting with mammalian chromatin (64).

In summary, this study demonstrates that activated pDCs respond to whole apoptotic cells, secreting IL-10, IL-6, or IFN-α depending on the coactivating pDC stimulus. Understanding how activated pDCs respond to apoptotic cells could potentially lead to new selective therapeutic targets to reduce aberrant inflammation in autoimmune diseases or promote immune reactivity to tumor-associated apoptotic cells.

## Author Contributions

JS, KM, MT, and RM performed experiments; JS analyzed results and generated the figures; MG designed the research; MG and JS wrote the paper.

## Conflict of Interest Statement

The authors declare that the research was conducted in the absence of any commercial or financial relationships that could be construed as a potential conflict of interest.
