# Supplementary figures and images for: Plasmacytoid Dendritic Cells Respond Directly to Apoptotic Cells by Secreting Immune Regulatory IL-10 or IFN-α
Source: Front Immunol. 2016 Dec 14;7:590. doi: 10.3389/fimmu.2016.00590 (PMC5155015; doi:10.3389/fimmu.2016.00590)

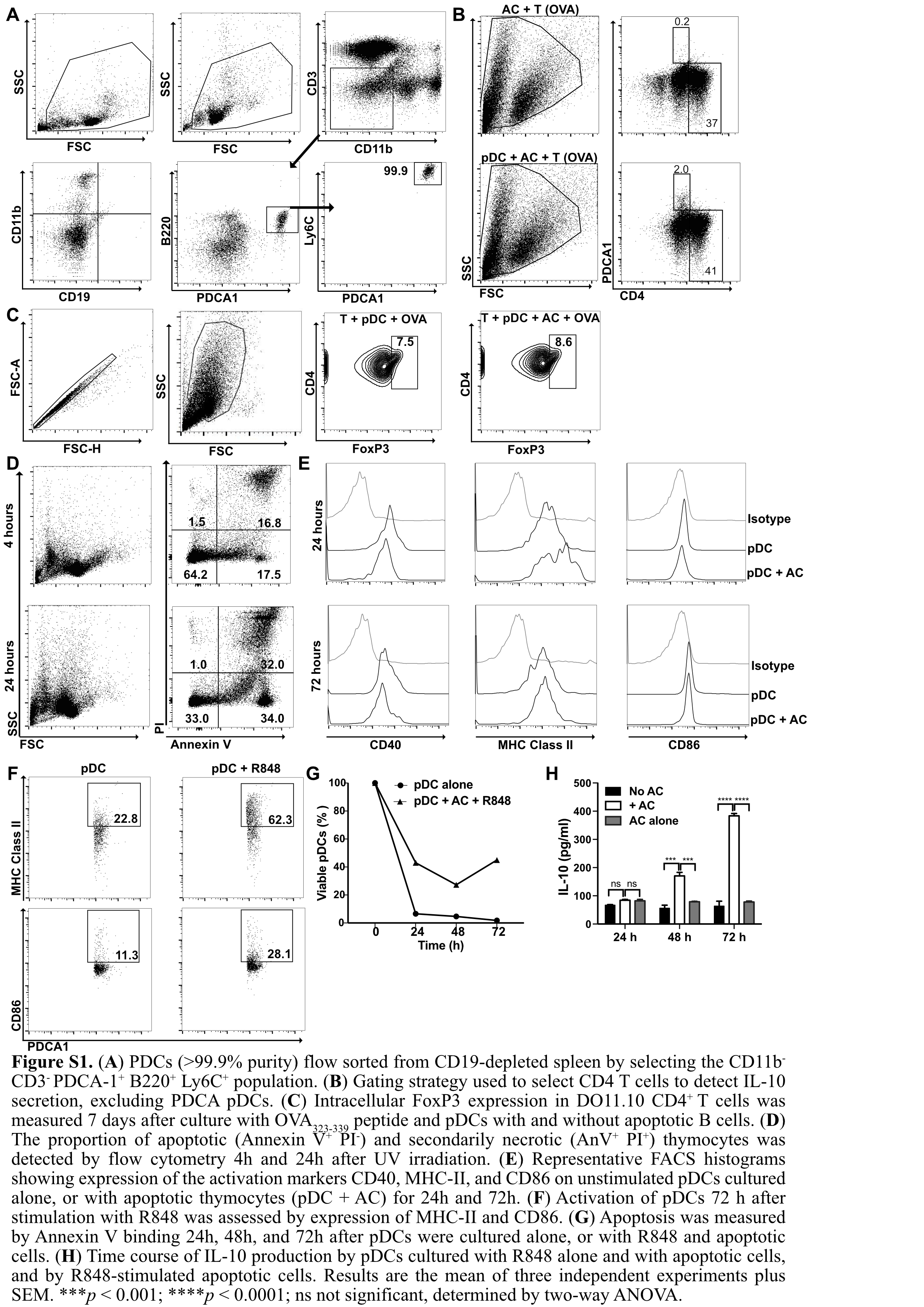

Supplement: Supplementary file 1 [file Image_1.JPEG]

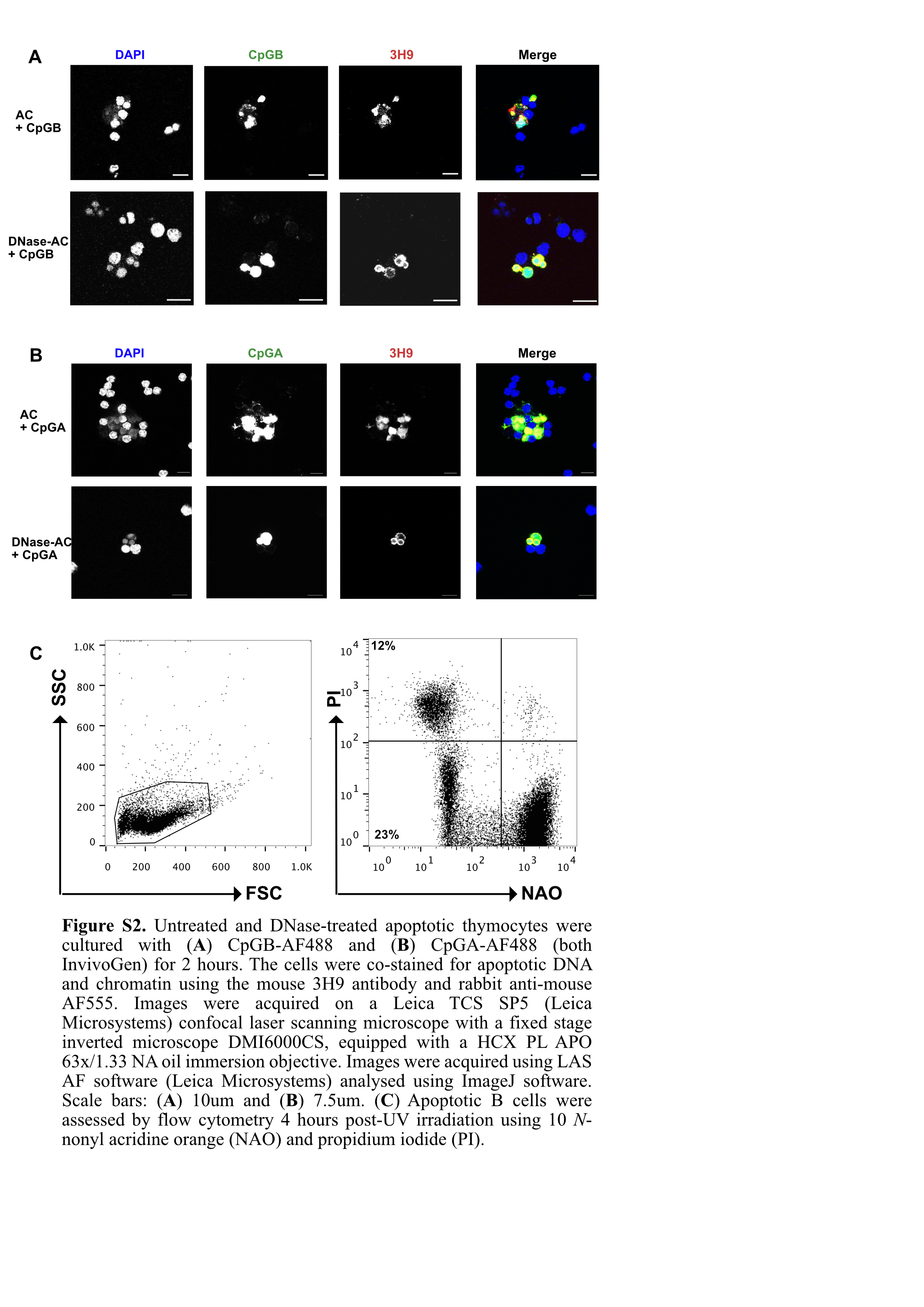

Supplement: Supplementary file 2 [file Image_2.jpg]
